# 2020 Editor's Choice Articles From JGMS: Focus on Vulnerable Populations

**DOI:** 10.1093/geroni/igab046.1325

**Published:** 2021-12-17

**Authors:** Lewis Lipsitz, Tamara Baker

## Abstract

This symposium will present four 2020 “Editor’s Choice” articles from the Journal of Gerontology Medical Sciences that focus on issues relevant to vulnerable older populations. Justin Golub and colleagues, in their article “Audiometric Age-Related Hearing Loss and Cognition in the Hispanic Community Health Study”, broaden the scope of age-related studies on audiometric hearing loss by using a large Hispanic cohort, a community largely excluded from previous hearing loss studies. By examining audiometrically-defined hearing loss and cognitive measures, Golub found links between hearing loss and lower neurocognition. Janice Atkins and colleagues, in “Preexisting Comorbidities Predicting COVID-19 and Mortality in the UK Biobank Community Cohort”, challenge the practice of simple age-based targeting of older adults to prevent severe COVID-19 infections, and show that specific high-risk comorbidities are better indicators of hospitalization and mortality. “Comparison of Recruitment Strategies for Engaging Older Minority Adults: Results from Take Heart”, by Jessica Ramsay and colleagues, examines methods used to recruit older adults of color from primarily low socio-economic households for behavioral and clinical health research. Ryon Cobb and coauthors, in their article “Self-reported Instances of Major Discrimination, Race/Ethnicity, and Inflammation among Older Adults: Evidence from the Health and Retirement Study”, investigate whether self-reported lifetime discrimination is a psychosocial factor influencing inflammation in older adults. Tamara Baker, the discussant, will highlight commonalities and lessons learned from these studies, including links between racial, socio-economic, or disease-related vulnerabilities of older adults and their health status, as well as best practices to account for these factors in future clinical trials.

